# Utilising VISULYZE-Generated Nomograms and OcuLign Alignment Tools to Improve Keratorefractive Lenticule Extraction Outcomes

**DOI:** 10.3390/jcm15093389

**Published:** 2026-04-29

**Authors:** Sharita Rosalyne Siregar, Lily Silva Ardiani, Johan Arif Hutauruk

**Affiliations:** JEC Eye Hospitals and Clinics, Jakarta 11520, Indonesia; doktertasha@gmail.com (S.R.S.); johan.hutauruk@jec.co.id (J.A.H.)

**Keywords:** nomogram, lenticule extraction, KLEx, VISULYZE, OcuLign, outcomes

## Abstract

**Background**: The keratorefractive lenticule extraction (KLEx) procedure has gained popularity because of its safety and effectiveness; however, its predictability remains variable, as it may be over- or under-corrected. This study aimed to evaluate visual and refractive outcomes following the utilisation of VISULYZE-generated nomograms (Carl Zeiss Meditec AG, Jena, Germany) and OcuLign cyclotorsion alignment tools (Carl Zeiss Meditec AG, Jena, Germany). **Methods**: This retrospective consecutive cohort study included patients undergoing KLEx, grouped into four sequential treatment phases: PRE-NOMOGRAM, NOMOGRAM, OCULIGN & NOMOGRAM, and OCULIGN. **Results**: A total of 688 patients (1264 eyes) were included. The OCULIGN group showed numerically higher efficacy, with 83.0% achieving post-operative (PO) uncorrected distance visual acuity (UDVA) 20/20 or better, with no loss of corrected distance visual acuity (CDVA) lines, and 67.0% gaining one line. Predictability and accuracy were high across groups, with the OCULIGN group demonstrating a strong correlation between attempted and achieved spherical equivalent (R^2^ = 0.9908), and 95.5% of eyes within ±0.50 D. Early PO outcomes suggested minimal refractive shift at one month. The NOMOGRAM group demonstrated numerically improved astigmatism correction (100.0% within ≤0.50 D), while the OCULIGN & NOMOGRAM group showed high precision in axis alignment. However, baseline imbalances were present, and between-group differences were relatively small. **Conclusions**: The use of OcuLign cyclotorsion alignment and VISULYZE-generated nomograms was associated with favourable visual and refractive outcomes following KLEx. However, given the retrospective, sequential design, imbalanced baseline, and limited follow-up duration, these findings should be interpreted cautiously. Further prospective, randomised studies with longer follow-up are required to confirm these observations.

## 1. Introduction

Keratorefractive lenticule extraction (KLEx) has gained popularity due to its safety and effectiveness [1,2,3,4,5,6]. However, its predictability regarding visual and refractive outcomes remains debated, as it may be over- or under-corrected in highly myopic astigmatism patients [6,7,8,9,10,11]. Unlike laser-assisted in situ keratomileusis (LASIK), KLEx devices lack a pupil tracking system to ensure proper centration, and no enhancement treatment is available to improve the results of KLEx procedures [1,7,8,9,10]. Therefore, it is essential to improve the predictability of the KLEx procedure to achieve optimal refractive outcomes.

Nomograms are efficient tools for improving the predictability of refractive procedures without compromising safety or efficacy [12,13,14]. Some recent studies have proposed linear regression formula-based [6,15,16] and AI-based nomograms [17,18,19]. To our knowledge, a universal standardised nomogram for the KLEx procedure is not currently available. The VISULYZE-generated nomogram (Carl Zeiss Meditec AG, Jena, Germany) and OcuLign cyclotorsion alignment tools (Carl Zeiss Meditec AG, Jena, Germany) were recently introduced for the VISUMAX800 femtosecond laser (Carl Zeiss Meditec AG, Jena, Germany) to improve the outcomes of the KLEx procedure [20,21,22,23]. An integrated iris image between IOLMaster700 (Carl Zeiss Meditec AG, Jena, Germany) and OcuLign helps increase accuracy in docking, suction, and laser treatment during the KLEx procedure [20,21,22,23]. In this study, we evaluated VISULYZE-generated nomograms and OcuLign cyclotorsion alignment tools to optimise visual and refractive outcomes in terms of efficacy, safety, predictability, accuracy, stability, astigmatism correction, and axis alignment in KLEx patients.

## 2. Materials and Methods

### 2.1. Study Design

This was a retrospective, consecutive cohort study with sequential treatment phases of patients who underwent KLEx procedures using a VISUMAX800 femtosecond laser under topical anaesthesia at JEC Eye Hospitals and Clinics in Jakarta, Indonesia. Ethical approval was obtained from the Medical and Health Research Ethics Committee of the Faculty of Medicine, Public Health, and Nursing of Universitas Gadjah Mada–Dr. Sardjito General Hospital (Ref. No: KE/FK/0161/EC/2025), and this study adhered to the tenets of the Declaration of Helsinki.

### 2.2. Participants

Inclusion Criteria: All patients who underwent the KLEx procedure using a VISUMAX800 femtosecond laser, performed by two eye surgeons, between October 2023 and February 2025, were included. We included all grades of myopia, which are defined as mild (up to −3.00 D), moderate (from −3.00 to −6.00 D), and high myopia (greater than −6.00 D); and all grades of astigmatism, mild (<1.00 D), moderate (from 1.00 to 2.00 D), severe (from 2.00 to 3.00 D), and extreme (greater than 3.00 D).

Exclusion Criteria: We excluded patients with ocular conditions, including refractive amblyopia, corneal scars, strabismus, and glaucoma.

### 2.3. Follow-Up

Our study consisted of three phases: pre-nomogram (October 2023 to April 2024), nomogram (April 2024 to October 2024), and oculign (November 2024 to February 2025). Patients were followed up at three consecutive post-operative (PO) visits: day one (PO1), one week (PO2), and one month (PO3).

### 2.4. Study Outcomes and Assessment

Patient data were obtained from hospital electronic medical records, including age, gender, and laterality (right eye (RE); left eye (LE)). Pre-operative (PreOp) examinations, including uncorrected distance visual acuity (UDVA), corrected distance visual acuity (CDVA) with subjective refraction (spherical (SPH), cylindrical (CYL), and axis), pachymetry, flap thickness, and residual stromal thickness (RST) using SCHWIND ORK-CAM (SCHWIND eye-tech-solutions GmBH & Co. KG, Kleinostheim, Germany), and pupil size using IOLMaster700, were gathered.

Intra-operative parameters were extracted from the VISUMAX800 femtosecond laser treatment profile, including intended correction (SPH, CYL, and axis), laser settings, namely, used nomogram, SPH and CYL adjustment, laser setting for SPH and CYL, cap thickness, optical zone of lenticule, centration target position x (PX) and y (PY), laser treatment centration type, achieved centration x (AX) and y (AY), decentring, energy index, spot and track distance, and cyclotorsion adjustment. OcuLign provided detected cyclotorsion angle and confidence level of detection data during the KLEx procedure. Laser treatment centration type was divided into corneal vertex and pupillary axis centration based on the laser treatment profile. Pupillary axis centration is defined by 0 degrees in PX, PY, AX, and AY, whereas the other is defined as corneal vertex centration. PO data were gathered during three consecutive follow-up visits (PO1, PO2, and PO3), including UDVA, CDVA, and subjective refraction: SPH, CYL, and axis.

The collected data were entered into an MS Excel spreadsheet. In the pre-nomogram phase, after collecting all PreOp and intra-operative data, we input them into VISULYZE 1.1. software to create nomograms. After generating nomograms, a look-up table containing adjusted SPH/CYL correction laser was downloaded (Table S1). The nomograms were then incorporated into the VISUMAX800 femtosecond laser during the KLEx procedure. The adjusted SPH/CYL correction laser was automated following the nomogram. This was the nomogram phase. Meanwhile, during the oculign phase, we divided into OCULIGN & NOMOGRAM, and OCULIGN groups. The nomogram used in the OCULIGN & NOMOGRAM group was the same as the one used in the nomogram phase. Therefore, there were four treatment groups in this study: PRE-NOMOGRAM, NOMOGRAM, OCULIGN & NOMOGRAM, and OCULIGN.

We present the study findings, including efficacy and safety of visual acuity, predictability and accuracy of spherical equivalent (SE) refraction, and astigmatism refraction (refractive astigmatism, target-induced (TIA) versus surgically induced astigmatism (SIA), and angle of error), based on Nine Standard Graphs [24] and double-angle astigmatism vector [25,26] using VISULYZE software.

### 2.5. Statistical Analysis

Statistical analysis was done using SPSS Statistics for Mac version 25 (SPSS Inc., Chicago, IL, USA). Descriptive statistics were used to present continuous variables as means ± standard deviations (SD), and categorical variables as frequencies and percentages. Pearson’s Chi-square and Kruskal–Wallis tests were performed to analyse categorical and continuous variables in the baseline characteristics of treatment groups, respectively. A bivariate correlation analysis using Pearson’s correlation was conducted between PreOp, intra-operative parameters, and PO outcomes. A multiple linear regression analysis was also performed to evaluate the association of any confounding factors and PO outcomes. A *p*-value of <0.05 was considered statistically significant.

## 3. Results

A total of 688 patients (1264 eyes) were included in this study. In the PRE-NOMOGRAM group, 191 patients (378 eyes) were included; in the NOMOGRAM group, 230 patients (425 eyes) were included. The OCULIGN & NOMOGRAM group comprised 122 patients (212 eyes), while the OCULIGN group consisted of 145 patients (249 eyes). Female patients predominated the overall cohort (374, 54.4%). Moderate myopia (600, 47.5%) and moderate astigmatism (515, 40.7%) were the majority. PreOp parameters, including CYL (*p* < 0.001), axis (*p* < 0.001), and flap thickness (*p* = 0.023), showed a statistically significant difference between treatment groups (Table 1).

Intra-operative parameters, including optical zone of lenticule (*p* < 0.001), decentring (*p* < 0.001), energy index (*p* < 0.001), spot distance (*p* < 0.001), track distance (*p* < 0.001), SPH adjustment (*p* < 0.001), CYL adjustment (*p* < 0.001), and OcuLign confidence interval (*p* = 0.005), demonstrated statistically significant differences between the groups. The detailed intra-operative parameters of the patients are presented in Table S2.

**Figure 1:**
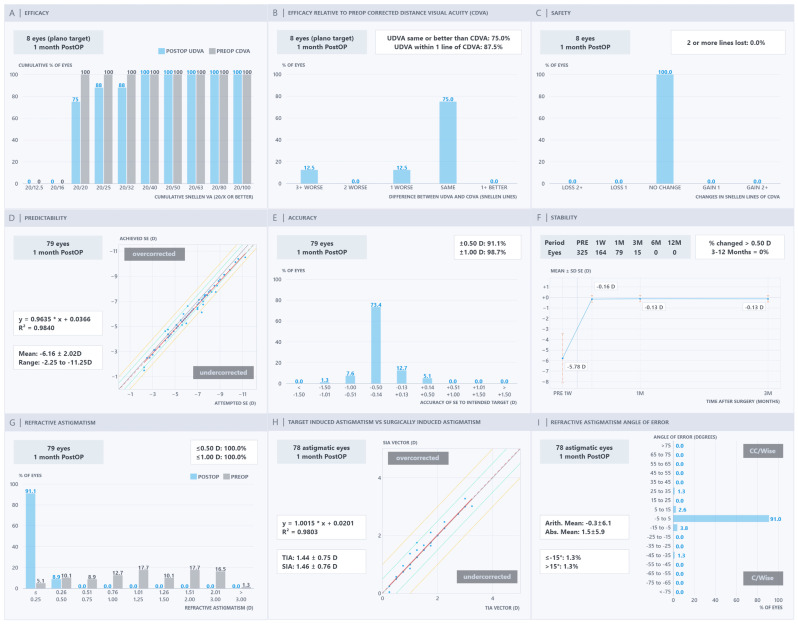
PRE-NOMOGRAM refractive outcomes: (**A**) efficacy, (**B**) efficacy relative to pre-operative corrected distance visual acuity, (**C**) safety, (**D**) predictability, (**E**) accuracy, (**F**) stability, (**G**) refractive astigmatism, (**H**) target-induced astigmatism versus surgically induced astigmatism, and (**I**) refractive astigmatism angle of error.

**Figure 2:**
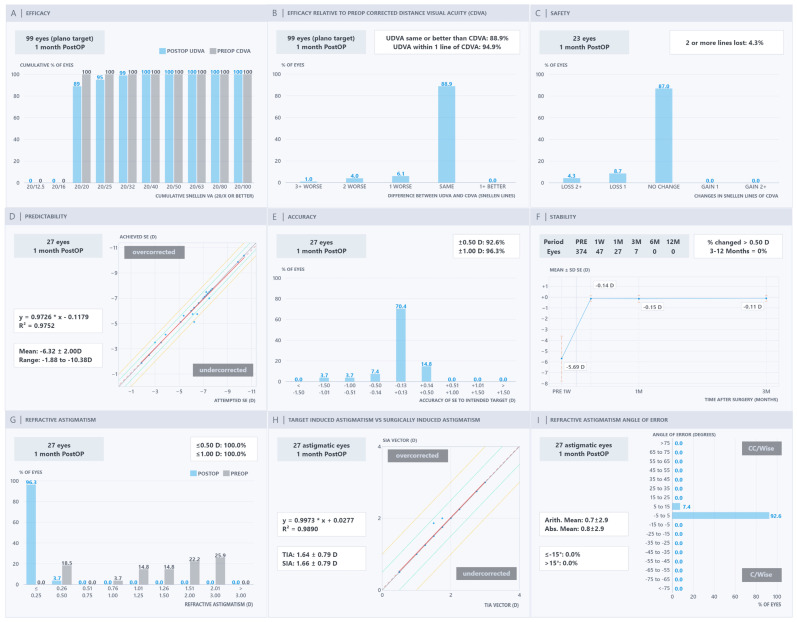
NOMOGRAM refractive outcomes: (**A**) efficacy, (**B**) efficacy relative to pre-operative corrected distance visual acuity, (**C**) safety, (**D**) predictability, (**E**) accuracy, (**F**) stability, (**G**) refractive astigmatism, (**H**) target-induced astigmatism versus surgically induced astigmatism, and (**I**) refractive astigmatism angle of error.

**Figure 3:**
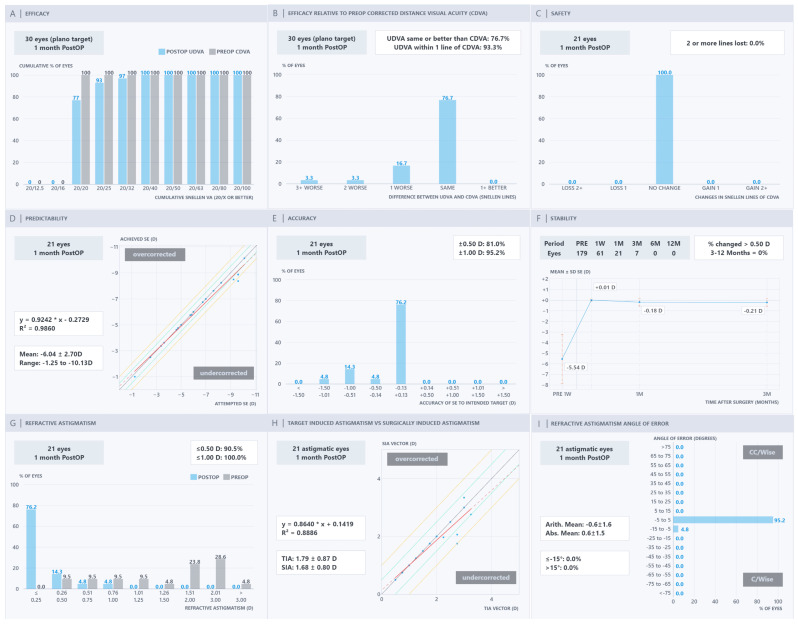
OCULIGN & NOMOGRAM refractive outcomes: (**A**) efficacy, (**B**) efficacy relative to pre-operative corrected distance visual acuity, (**C**) safety, (**D**) predictability, (**E**) accuracy, (**F**) stability, (**G**) refractive astigmatism, (**H**) target-induced astigmatism versus surgically induced astigmatism, and (**I**) refractive astigmatism angle of error.

**Figure 4:**
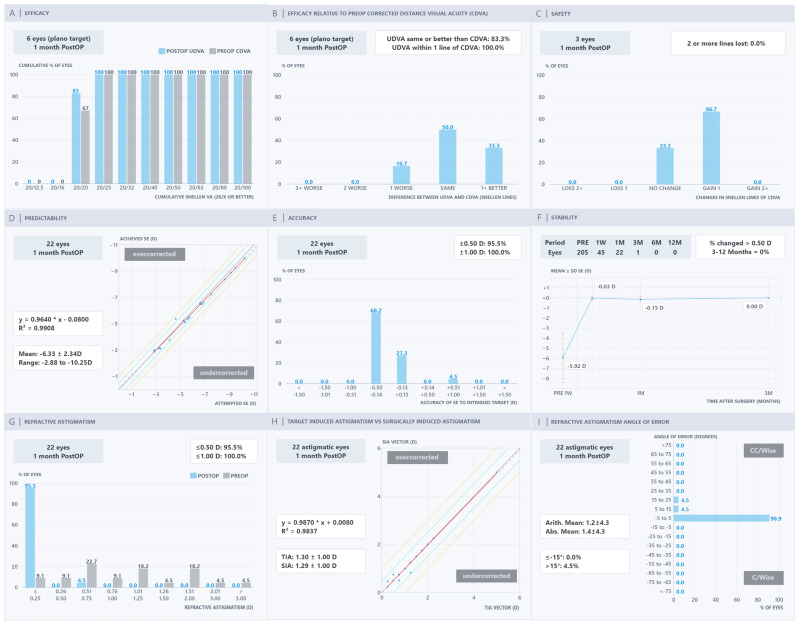
OCULIGN refractive outcomes: (**A**) efficacy, (**B**) efficacy relative to pre-operative corrected distance visual acuity, (**C**) safety, (**D**) predictability, (**E**) accuracy, (**F**) stability, (**G**) refractive astigmatism, (**H**) target-induced astigmatism versus surgically induced astigmatism, and (**I**) refractive astigmatism angle of error.

The Nine Standard Graphs of the PRE-NOMOGRAM, NOMOGRAM, OCULIGN & NOMOGRAM, and OCULIGN groups are presented in Figure 1, Figure 2, Figure 3 and Figure 4. In terms of efficacy, the OCULIGN group demonstrated higher proportions of favourable outcomes: 83.0% of eyes showed PO 20/20 UDVA equal to or better than PreOp CDVA, and 100.0% of patients achieved PO UDVA within one line of PreOp CDVA. Regarding safety, no eyes in the OCULIGN group lost a line of CDVA, and two-thirds gained one line. The OCULIGN group also demonstrated high predictability, with a slope of 0.9640 and a strong correlation coefficient (R^2^ = 0.9908) between attempted and achieved SE. Refractive accuracy was similarly high, with 95.5% of eyes within ±0.50 D of intended correction. Early PO assessment suggested minimal refractive shift at one month. As for astigmatism outcomes, the NOMOGRAM group demonstrated higher rates of correction, with 100.0% of patients within ±0.50 D, achieving a strong vector match (slope of 0.9973; R^2^ = 0.9890). The OCULIGN group achieved a correction index (SIA/TIA) close to unity (0.992), indicating a high degree of proportional correction. Regarding axis alignment, the OCULIGN & NOMOGRAM group demonstrated high precision, with 95.2% eyes within ±5 degrees and a mean absolute error of 0.6 degrees. Overall, while differences among the four treatment groups were observed, these findings should be interpreted cautiously, given baseline differences. Detailed comparisons are provided in Table 2.

Double-angle astigmatism vector plots showed differences in the distribution and clustering of residual astigmatism across treatment groups (Figure S1). The PRE-NOMOGRAM group showed a wider dispersion of data points, indicating greater variability in astigmatism outcomes. The NOMOGRAM group demonstrated a tighter clustering around the origin, suggesting an improved magnitude of astigmatism correction. The OCULIGN group showed a more symmetric vector distribution, consistent with improved axis alignment. Meanwhile, the OCULIGN & NOMOGRAM group showed a more compact clustering with reduced dispersion, suggesting improved overall vector accuracy. Vector analysis further demonstrated that the correction index in the OCULIGN group was 0.992, indicating a trend toward values closer to unity. Axis alignment also appeared more precise in the OCULIGN group, as reflected by reduced angular dispersion. However, these observations are descriptive, and no further statistical comparisons of the vector parameters were performed.

Significant correlations were observed between PO SPH and PreOp parameters, including age (r = −0.242; *p* = 0.001), myopia grade (r = −0.174; *p* = 0.015), and SPH (r = 0.195; *p* = 0.007). Whereas a strong association with PO axis was observed only in pachymetry (r = −0.193; *p* = 0.007), no correlation was observed between factors associated with PO CYL. Meanwhile, PO UDVA was associated with myopia grade (r = −0.123; *p* = 0.019), PreOp SPH (r = 0.153; *p* = 0.004), and UDVA (r = 0.106; *p* = 0.045). A strong correlation was also found between age and myopia (r = 0.072; *p* = 0.010) and astigmatism grades (r = −0.088; *p* = 0.002). However, no significant correlations were found between treatment group and PO outcomes, nor between intra-operative parameters, including laser energy, optical zone, and refractive outcomes.

Multiple linear regression analysis was performed to evaluate factors associated with PO outcomes. Treatment group, PreOp SPH, CYL, axis, UDVA, CDVA, age, and pachymetry were included in the analysis. These variables showed a statistically significant association with PO SPH (F (8, 185) = 2.284; *p* = 0.024; R^2^ = 0.090). However, no significant associations were observed for PO CYL (F (8, 185) = 1.052; *p* = 0.339; R^2^ = 0.043), axis (F (8, 185) = 1.689; *p* = 0.104; R^2^ = 0.068), UDVA (F (8, 351) = 1.478; *p* = 0.164; R^2^ = 0.033), and CDVA (F (8, 185) = 0.915; *p* = 0.505; R^2^ = 0.038). We also found that the treatment group was not independently associated with PO outcomes after adjustment (*p* = 0.858), suggesting that the observed differences between groups may be influenced by the variability in baseline characteristics and confounding factors rather than the interventions themselves. The relatively low R^2^ values indicate that only a small proportion of the variability in PO outcomes is explained by the included variables, suggesting that additional unmeasured factors may contribute to refractive outcomes.

## 4. Discussion

There are some disputes regarding the KLEx procedure’s predictability in visual and refractive outcomes, as it may be over- or under-corrected, especially in high myopic patients [6,7,8,9,10,27,28,29]. There is a need to improve the predictability of the KLEx procedure to achieve optimal refractive outcomes. Nomograms are considered reliable prediction tools [30,31], and linear regression formula-based nomograms [6,15,16], as well as AI-based nomograms, have been proposed recently [17,18,19]. To our knowledge, this is the first study to evaluate the use of VISULYZE-generated nomograms and OcuLign cyclotorsion alignment tools to enhance visual and refractive outcomes in KLEx patients. We found that OcuLign cyclotorsion alignment demonstrated numerically higher proportions of favourable outcomes in efficacy, safety, predictability, accuracy, and stability. Meanwhile, the VISULYZE-generated nomogram tools showed numerically higher proportions of favourable enhancement in astigmatism correction, TIA/SIA vector match, and axis alignment in KLEx patients.

Several factors can influence visual and refractive outcomes after laser vision correction, including age, sphere and astigmatism degrees, and other PreOp parameters [32,33,34,35]. Our study found a strong correlation between age and PO SPH (*p* = 0.001). Recent studies have shown that age contributes to refractive outcomes [18,36,37,38], findings similar to ours. Older patients experience more refractive changes than younger patients [37]. We found that myopia grade was also correlated with PO UDVA (*p* = 0.019) and SPH (*p* = 0.015). These findings aligned with previous research indicating that the degree of myopia can affect refractive outcomes [18,36,37,38]. They also noted that higher grades of myopia are associated with greater under-correction results [18]. Besides myopia grade, recent studies also reported that laser energy and the diameter of the optical zone can influence refractive outcomes [18,36,37,38]. Low laser energy may contribute to under-correction results, but it can also lead to faster visual recovery after the KLEx procedure [18,36]. However, in the current study, no significant correlation was found between laser energy and refractive outcomes. Additionally, we found no significant correlation between optical zones and PO results, which differed from a previous study by Liu et al. [38], who reported that a larger optical zone results in more over-correction than a smaller one. Treatment centration showed a strong correlation with intra-operative decentring (*p* < 0.001), and our result was also in line with recent studies [39,40,41,42]. However, since our central vertex centration dominated the cohorts, further studies focusing on pupillary axis centration are needed.

In the current study, we observed that the OCULIGN group demonstrated higher proportions of favourable efficacy, safety, predictability, accuracy, and stability. The OCULIGN & NOMOGRAM group showed higher proportions of favourable axis alignment. Meanwhile, the NOMOGRAM group represented higher proportions of favourable astigmatism correction and TIA/SIA vector match. Combining refractive astigmatism correction, vector match, and axis alignment using the nomogram demonstrated a trend toward improvement, as shown in the NOMOGRAM and OCULIGN & NOMOGRAM groups. However, our findings should be interpreted with caution due to the differences in baseline patient characteristics.

Compared with previously proposed AI-based nomograms, the VISULYZE-generated nomogram combines linear regression with a machine learning algorithm [43]. Cui et al. [18] revealed that machine learning-based nomograms had similar safety, better efficacy, and comparable predictability to surgeon-based nomograms. Meanwhile, Park et al. [19] compared various machine learning algorithms, and AdaBoost achieved the highest predictive performance compared to multiple linear regression, decision tree, XGBoost, and multilayer perceptron.

The limitations of this study were that it was conducted at a single centre of a tertiary eye hospital in Jakarta, Indonesia. Despite the fact that there were four treatment groups corresponding to three consecutive phases (pre-nomogram, nomogram, and oculign), our study was not a randomised controlled trial. The treatment groups corresponded to sequential periods, and temporal confounding, including surgeon learning curve with OcuLign cyclotorsion alignment tools throughout the study period, different patient selection, which showed in the imbalance group size, and secular trends, cannot be fully separated from treatment effects. These factors could also have influenced the results, contributing to temporal and patient selection bias. Furthermore, given its retrospective nature, there is also a risk of selection, data, and reporting bias in this study. The SPH and CYL powers of our patients ranged from 0.00 D to −10.75 D and 0.00 D to −3.25 D, respectively. Therefore, our nomogram can only be used in patients with SPH ≤ −10.75 and CYL ≤ −3.25. A broader range in SPH and CYL power is needed in the future. The imbalance in the distribution of astigmatism patients across treatment groups also contributed to our results. Furthermore, a longer PO follow-up is also needed. Since our patients also come from outside Jakarta, a mandatory follow-up visit for more than 6 months can be impractical. Refractive stability cannot be definitively assessed without a longer follow-up period (more than 3–6 months); therefore, the stability of our findings should be interpreted cautiously. Future studies should aim to be prospective, use a randomised design, and have a longer follow-up period; direct comparison with AI-based nomograms is also required.

Our hospital is one of the tertiary eye hospitals in Jakarta, Indonesia, with experienced surgeons who have performed over 25,000 KLEx procedures. Although this nomogram is based on the expertise of two senior ophthalmologists specialising in cornea, cataract, and refractive surgery, it has shown promising results in improving KLEx outcomes. Furthermore, the R^2^ value for the NOMOGRAM was 0.9752, indicating more favourable predictive performance than in the previous survey [14]. However, the R^2^ value should be interpreted cautiously, particularly with small sample sizes. Hopefully, our nomogram can be implemented and add value to daily clinical practice, especially in KLEx centres that currently lack such nomogram tools.

## 5. Conclusions

In conclusion, in this retrospective sequential cohort study, the implementation of OcuLign cyclotorsion alignment and VISULYZE-generated nomograms was associated with favourable refractive outcomes in myopic and astigmatic patients following KLEx procedures. However, given the non-randomised study design, limited follow-up duration, and potential confounding factors, these findings should be interpreted with caution, especially regarding refractive stability. Further prospective, randomised studies with longer follow-up are warranted to confirm these results.

## Figures and Tables

**Table 1:** Baseline characteristics and pre-operative parameters.

Details	PRE-NOMOGRAM (N = 191 Patients, 100%)	NOMOGRAM (N = 230 Patients, 100%)	OCULIGN & NOMOGRAM (N = 122 Patients, 100%)	OCULIGN (N = 145 Patients, 100%)	*p*-Value
Gender					0.335 ^a^
Male	84 (44.0%)	97 (42.2%)	59 (48.4%)	74 (51.0%)	
Female	107 (56.0%)	133 (57.8%)	63 (51.6%)	71 (49.0%)	
Age (years)	Mean 26.67 ± SD 8.45 (Range 17–50)	Mean 27.62 ± SD 8.32 (Range 17–51)	Mean 26.26 ± SD 8.71 (Range 17–50)	Mean 26.81 ± SD 9.26 (Range 17–56)	0.052 ^b^
Laterality					<0.001 ^a,^*
Unilateral	6 (3.1%)	5 (2.2%)	32 (26.2%)	41 (28.3%)	
Bilateral	185 (96.9%)	225 (97.8%)	90 (73.8%)	104 (71.7%)	
**Details**	**PRE-NOMOGRAM** **(N = 378 Eyes, 100%)**	**NOMOGRAM** **(N = 425 Eyes, 100%)**	**OCULIGN** **& NOMOGRAM** **(N = 212 Eyes, 100%)**	**OCULIGN** **(N = 249 eyes, 100%)**	***p*-Value**
Eye					0.521 ^a^
RE	193 (51.1%)	212 (49.9%)	108 (50.9%)	113 (45.4%)	
LE	185 (48.9%)	213 (50.1%)	104 (49.1%)	136 (54.6%)	
Centration					<0.001 ^a,^*
Vertex	342 (90.5%)	417 (98.1%)	212 (100.0%)	246 (98.8%)	
Pupil	36 (9.5%)	6 (1.4%)	0	3 (1.2%)	
N/A	0	2 (0.5%)	0	0	
Myopia grade					0.002 ^a,^*
Mild	91 (24.1%)	74 (17.4%)	60 (4.7%)	75 (30.1%)	
Moderate	181 (47.9%)	225 (52.9%)	96 (45.3%)	98 (39.4%)	
High	106 (28.0%)	126 (29.6%)	56 (26.4%)	76 (30.5%)	
Astigmatism grade					<0.001 ^a,^*
Mild	107 (8.5%)	145 (11.5%)	89 (7.0%)	78 (6.2%)	
Moderate	165 (13.1%)	188 (14.9%)	77 (6.1%)	85 (6.7%)	
Severe	79 (6.3%)	84 (6.6%)	44 (3.5%)	61 (4.8%)	
Extreme	27 (2.1%)	8 (0.6%)	2 (0.2%)	25 (2.0%)	
**PreOp** **Parameters**	**PRE-NOMOGRAM** **(N = 378 Eyes, 100%)**	**NOMOGRAM** **(N = 425 Eyes, 100%)**	**OCULIGN** **& NOMOGRAM** **(N = 212 Eyes, 100%)**	**OCULIGN** **(N = 249 Eyes, 100%)**	***p*-Value**
SPH	−4.80 (−0.25 to −10.50)	−5.05 (−0.25 to −9.50)	−4.66 (0.00 to −9.50)	−4.70 (0.00 to −10.75)	0.136 ^b^
CYL	−1.49 (0.00 to −5.00)	−1.28 (0.00 to −4.00)	−1.21 (−0.25 to −3.25)	−1.58 (0.00 to −5.00)	<0.001 ^b,^*
AX	76.90 (0–180)	121.40 (0–180)	120.60 (5–180)	112.21 (0–180)	<0.001 ^b,^*
Pachymetry	550.61 ± 34.692 (408–667)	549.68 ± 33.349 (476–666)	550.09 ± 30.543 (484–641)	550.55 ± 32.958 (463–664)	0.959 ^b^
Flap thickness	108.47 ± 3.609 (100–110)	108.84 ± 3.204 (100–110)	109.15 ± 2.794 (100–110)	108.31 ± 3.752 (100–110)	0.024 ^b,^*
RST	349.03 ± 46.039 (131–494)	346.40 ± 40.665 (108–494)	352.58 ± 50.230 (124–495)	348.08 ± 42.357 (273–471)	0.702 ^b^
Pupil size	5.153 ± 1.2308 (2.5–8.4)	5.347 ± 1.2468 (2.7–9.2)	5.324 ± 1.0838 (3.0–7.6)	5.389 ± 1.2558 (2.8–9.0)	0.102 ^b^

* Statistically significant, *p*-value < 0.05. ^a^ Using Pearson Chi-Square test; ^b^ Kruskal–Wallis test. LE, left eye; N/A, not available; PreOp, pre-operative; RE, right eye; RST, residual stromal thickness.

**Table 2:** Detailed comparison of Nine Standard Graphs between treatment groups.

Parameter	PRE-NOMOGRAM	NOMOGRAM	OCULIGN & NOMOGRAM	OCULIGN
A. Efficacy	75.0% PO UDVA ≥ PreOp CDVA	89.0% PO UDVA ≥ PreOp CDVA	77.0% PO UDVA ≥ PreOp CDVA	83.0% PO UDVA ≥ PreOp CDVA
B. Efficacy	87.5% within 1 line PreOp CDVA	94.9% within 1 line PreOp CDVA	93.3% within 1 line PreOp CDVA	100.0% within 1 line PreOp CDVA
C. Safety	No loss of lines	4.3% 2 or more lines lost	No loss of lines	No loss of lines; 67% gained 1 line
D. Predictability	Slope 0.9635, R^2^ 0.9840	Slope 0.9726, R^2^ 0.9752	Slope 0.9242, R^2^ 0.9860	Slope 0.9640, R^2^ 0.9908
E. Accuracy	±0.50 D 91.1%	±0.50 D 92.6%	±0.50 D 81.0%	±0.50 D 95.5%
F. Stability	Minimal shift (−0.13 D)	Minimal shift (−0.11 D)	Slight myopic drift (−0.21 D)	No shift
G. Astigmatism Correction	≤0.50 D 100.0% (91.1% ≤ 0.25 D)	≤0.50 D 100.0% (96.3% ≤ 0.25 D)	≤0.50 D 90.5% (76.2% ≤ 0.25 D)	≤0.50 D 95.5% (95.5% ≤ 0.25 D)
H. Vector Match	Slope 1.0015, R^2^ 0.9803	Slope 0.9973, R^2^ 0.9890	Slope 0.8640, R^2^ 0.8886	Slope 0.987, R^2^ 0.9837
I. Correction Index (SIA/TIA)	1.46/1.44 = 1.013	1.66/1.64 = 1.012	1.68/1.79 = 0.938	1.29/1.30 = 0.992

CDVA, corrected distance visual acuity; D, dioptre; PO, post-operative; SIA, surgically induced astigmatism; TIA, target-induced astigmatism; UDVA, uncorrected distance visual acuity.

## Data Availability

The datasets generated and/or analysed during the current study are not publicly available due to confidential reasons but are available from the corresponding author on reasonable request.

## Supplementary Materials

The following supporting information can be downloaded at: https://www.mdpi.com/article/10.3390/jcm15093389/s1, Figure S1: Double-angle astigmatism vector: (A) PRE-NOMOGRAM, (B) NOMOGRAM, (C) OCULIGN & NOMOGRAM, and (D) OCULIGN; Table S1: Look-up table nomograms; Table S2: Intra-operative parameters.

## Abbreviations

The following abbreviations are used in this manuscript:

AX	achieved centration x
AY	achieved centration y
CDVA	corrected distance visual acuity
CYL	cylindrical
D	dioptre
KLEx	keratorefractive lenticule extraction
LASIK	laser-assisted in situ keratomileusis
LE	left eye
PreOp	pre-operative
PO	post-operative
PX	target position x
PY	target position y
RE	right eye
RST	residual stromal thickness
SD	standard deviations
SE	spherical equivalent
SIA	surgically induced astigmatism
SPH	spherical
TIA	target-induced astigmatism
UDVA	uncorrected distance visual acuity

## References

[1] Liu Q., Yang X., Lin L., Liu M., Lin H., Liu F., Xie Y., Lam D.S.C. (2019). Review on Centration, Astigmatic Axis Alignment, Pupil Size and Optical Zone in SMILE. Asia Pac. J. Ophthalmol..

[2] He M., Huang W., Zhong X. (2015). Central corneal sensitivity after small incision lenticule extraction versus femtosecond laser-assisted LASIK for myopia: A meta-analysis of comparative studies. BMC Ophthalmol..

[3] Li M., Li M., Chen Y., Miao H., Yang D., Ni K., Zhou X. (2019). Five-year results of small incision lenticule extraction (SMILE) and femtosecond laser LASIK (FS-LASIK) for myopia. Acta Ophthalmol..

[4] Lin F., Xu Y., Yang Y. (2014). Comparison of the visual results after SMILE and femtosecond laser-assisted LASIK for myopia. J. Refract. Surg..

[5] Zhang Y., Shen Q., Jia Y., Zhou D., Zhou J. (2016). Clinical Outcomes of SMILE and FS-LASIK Used to Treat Myopia: A Meta-analysis. J. Refract. Surg..

[6] Liang G., Zha X., Zhang F. (2014). An early clinical study on femtosecond small incision lenticule extraction for myopia and myopic astigmatism with different target refraction designs. Chin. J. Optom. Ophthalmol..

[7] Gimbel H.V., Stoll S.B. (2001). Photorefractive keratectomy with customized segmental ablation to correct irregular astigmatism after laser in situ keratomileusis. J. Refract. Surg..

[8] Grim M., Sheard J., Martin L. (2005). LASIK enhancement using excimer laser ablation on the back of the flap. J. Refract. Surg..

[9] Güell J.L., Lohmann C.P., Malecaze F.A., Junger J., Muller A., Deneuville S. (1999). Intraepithelial photorefractive keratectomy for regression after laser in situ keratomileusis. J. Cataract. Refract. Surg..

[10] Versace P., Watson S.L. (2005). Cornea-sparing laser in situ keratomileusis: Ablation on the flap. J. Cataract. Refract. Surg..

[11] Pietilä J., Huhtala A., Mäkinen P., Nättinen J., Rajala T., Salmenhaara K., Uusitalo H. (2018). Uncorrected visual acuity, postoperative astigmatism, and dry eye symptoms are major determinants of patient satisfaction: A comparative, real-life study of femtosecond laser in situ keratomileusis and small incision lenticule extraction for myopia. Clin. Ophthalmol..

[12] Mrochen M., Hafezi F., Iseli H.P., Löffler J., Seiler T. (2006). Nomograms for the improvement of refractive outcomes. Ophthalmologe.

[13] Saeed A. (2018). A novel refractive nomogram for the Custom-Q laser-assisted in-situ keratomileusis treatment of myopia. Egypt. J. Cataract. Refract. Surg..

[14] Liang G., Chen X., Zha X., Zhang F. (2017). A Nomogram to Improve Predictability of Small-Incision Lenticule Extraction Surgery. Med. Sci. Monit..

[15] Wu F., Yin H., Chen X., Yang Y. (2020). Investigation of predictability and influence factors of the achieved lenticule thickness in small incision lenticule extraction. BMC Ophthalmol..

[16] Yu N., Hou X., Liu C., Chen P., Ye Y., Lan M., Zhuang J., Yu K. (2025). A Nomogram to Improve the Predictability of High Myopic Astigmatism in Small Incision Lenticule Extraction Surgery. J. Refract. Surg..

[17] Luft N., Mohr N., Spiegel E., Marchi H., Siedlecki J., Harrant L., Mayer W.J., Dirisamer M., Priglinger S.G. (2024). Optimizing Refractive Outcomes of SMILE: Artificial Intelligence versus Conventional State-of-the-Art Nomograms. Curr. Eye Res..

[18] Cui T., Wang Y., Ji S., Li Y., Hao W., Zou H., Jhanji V. (2020). Applying Machine Learning Techniques in Nomogram Prediction and Analysis for SMILE Treatment. Am. J. Ophthalmol..

[19] Park S., Kim H., Kim L., Kim J.-k., Lee I.S., Ryu I.H., Kim Y. (2021). Artificial intelligence-based nomogram for small-incision lenticule extraction. Biomed. Eng. OnLine.

[20] Carl Zeiss AG ZEISS VISULYZE: Your All-In-One Refractive Data Reporting and Nomogram Tool. https://www.zeiss.com/meditec/en/products/refractive-lasers/zeiss-visulyze.html#.

[21] Carl Zeiss AG ZEISS VISUMAX 800. https://www.zeiss.com/meditec/en/products/refractive-lasers/femtosecond-laser-solutions/zeiss-visumax-800.html.

[22] Ganesh S., Sriganesh S.S., Karanam D. (2025). Visual and Refractive Outcomes of Small Incision Lenticule Extraction with VisuMax 800 in 1500 Eyes. J. Refract. Surg..

[23] Reinstein D.Z., Archer T.J., Potter J.G., Gupta R., Wiltfang R. (2023). Refractive and Visual Outcomes of SMILE for Compound Myopic Astigmatism with the VISUMAX 800. J. Refract. Surg..

[24] Reinstein D.Z., Archer T.J., Randleman J.B. (2014). JRS standard for reporting astigmatism outcomes of refractive surgery. J. Refract. Surg..

[25] Abulafia A., Koch D.D., Holladay J.T., Wang L., Hill W. (2018). Pursuing perfection in intraocular lens calculations: IV. Rethinking astigmatism analysis for intraocular lens-based surgery: Suggested terminology, analysis, and standards for outcome reports. J. Cataract. Refract. Surg..

[26] Kohnen T., Næser K., Holladay J.T., Stulting R.D., Wang L., Abulafia A., Koch D.D. (2025). Standards for analyzing astigmatic outcomes: Part I: Astigmatism basics. J. Cataract. Refract. Surg..

[27] Chan T.C., Ng A.L., Cheng G.P., Wang Z., Ye C., Woo V.C., Tham C.C., Jhanji V. (2016). Vector analysis of astigmatic correction after small-incision lenticule extraction and femtosecond-assisted LASIK for low to moderate myopic astigmatism. Br. J. Ophthalmol..

[28] Khalifa M.A., Ghoneim A.M., Shaheen M.S., Piñero D.P. (2017). Vector analysis of astigmatic changes after small-incision lenticule extraction and wavefront-guided laser in situ keratomileusis. J. Cataract. Refract. Surg..

[29] Zhang J., Wang Y., Wu W., Xu L., Li X., Dou R. (2015). Vector analysis of low to moderate astigmatism with small incision lenticule extraction (SMILE): Results of a 1-year follow-up. BMC Ophthalmol..

[30] Liang W., Zhang L., Jiang G., Wang Q., Liu L., Liu D., Wang Z., Zhu Z., Deng Q., Xiong X. (2015). Development and validation of a nomogram for predicting survival in patients with resected non-small-cell lung cancer. J. Clin. Oncol..

[31] Wen J., Ye F., He X., Li S., Huang X., Xiao X., Xie X. (2016). Development and validation of a prognostic nomogram based on the log odds of positive lymph nodes (LODDS) for breast cancer. Oncotarget.

[32] Artini W., Riyanto S.B., Hutauruk J.A., Gondhowiardjo T.D., Kekalih A. (2018). Predictive Factors for Successful High Myopia Treatment Using High-Frequency Laser-In-Situ Keratomileusis. Open Ophthalmol. J..

[33] Cao H., Zhang L., Liang S., Chen X., Jhanji V., Wang Y. (2022). Predictive factors of posterior corneal shift after small incision lenticule extraction: A 5-year follow-up study. Acta Ophthalmol..

[34] Zhang M., Ji S., Huo Y.A.N., Bai S., Tao Z., Zhang J., Cao H., Zou H., Zhao X., Wang Y.A.N. (2025). Analyzing the Effect of Surgical and Corneal Parameters on the Postoperative Refractive Outcomes of Smile in Myopic Eyes Based on Machine Learning. Am. J. Ophthalmol..

[35] Kim J.R., Hwang H.B., Mun S.J., Chung Y.T., Kim H.S. (2014). Efficacy, predictability, and safety of small incision lenticule extraction: 6-months prospective cohort study. BMC Ophthalmol..

[36] Donate D., Thaëron R. (2016). Lower Energy Levels Improve Visual Recovery in Small Incision Lenticule Extraction (SMILE). J. Refract. Surg..

[37] Rao S.N., Chuck R.S., Chang A.H., LaBree L., McDonnell P.J. (2000). Effect of age on the refractive outcome of myopic photorefractive keratectomy. J. Cataract. Refract. Surg..

[38] Liu M., Sun Y., Wang D., Zhang T., Zhou Y., Zheng H., Liu Q. (2015). Decentration of optical zone center and its impact on visual outcomes following SMILE. Cornea.

[39] Chan T.C.Y., Wan K.H., Kang D.S.Y., Tso T.H.K., Cheng G.P.M., Wang Y. (2019). Effect of corneal curvature on optical zone decentration and its impact on astigmatism and higher-order aberrations in SMILE and LASIK. Graefes Arch. Clin. Exp. Ophthalmol..

[40] Ding X., Fu D., Wang L., Zhou X., Yu Z. (2021). Functional Optical Zone and Visual Quality After Small-Incision Lenticule Extraction for High Myopic Astigmatism. Ophthalmol. Ther..

[41] Kang D.S.Y., Lee H., Reinstein D.Z., Roberts C.J., Arba-Mosquera S., Archer T.J., Kim E.K., Seo K.Y., Kim T.I. (2018). Comparison of the Distribution of Lenticule Decentration Following SMILE by Subjective Patient Fixation or Triple Marking Centration. J. Refract. Surg..

[42] Liu S., Zhang X., You Z., Zhou X. (2020). Comparison of the Distribution of Lenticule Decentration Following SMILE by Pupil Center or Tear Film Mark Centration. J. Refract. Surg..

[43] Wan Q., Wei R., Tang J., Deng Y.-P., Ma K. (2025). VISULYZE-Generated Nomogram-Assisted KLEx for Myopia and Astigmatism Correction: 3-Month Follow-Up Results. Clin. Ophthalmol..

