# Supplementary material for: Utilising VISULYZE-Generated Nomograms and OcuLign Alignment Tools to Improve Keratorefractive Lenticule Extraction Outcomes
Source: J Clin Med. 2026 Apr 29;15(9):3389. doi: 10.3390/jcm15093389 (PMC13163256; doi:10.3390/jcm15093389)
Supplement: Supplementary file 1 [file jcm-15-03389-s001.zip › Figure S1.pdf]

Figure S1. Double-angle astigmatism vector (A) PRE-NOMOGRAM

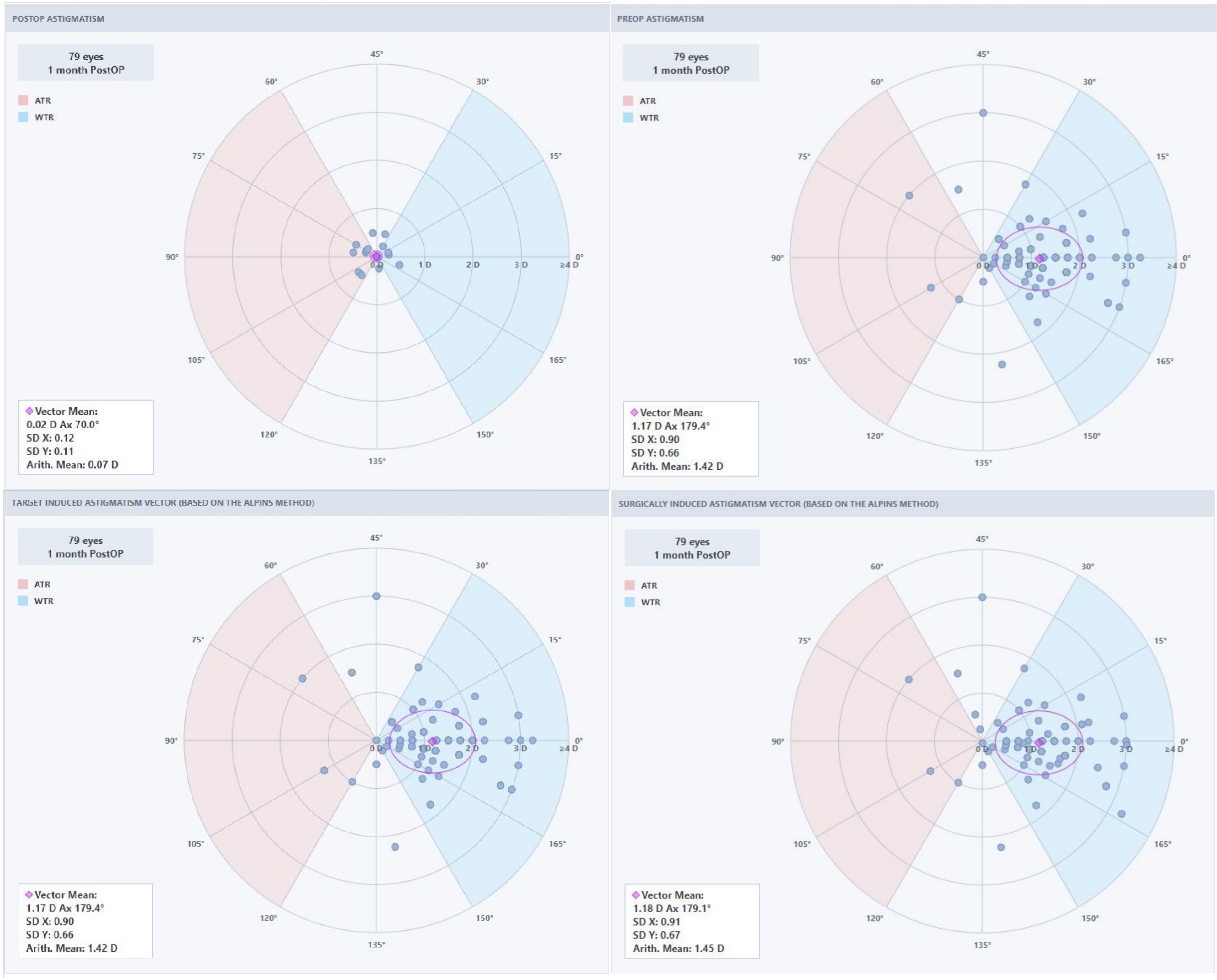

(B) NOMOGRAM

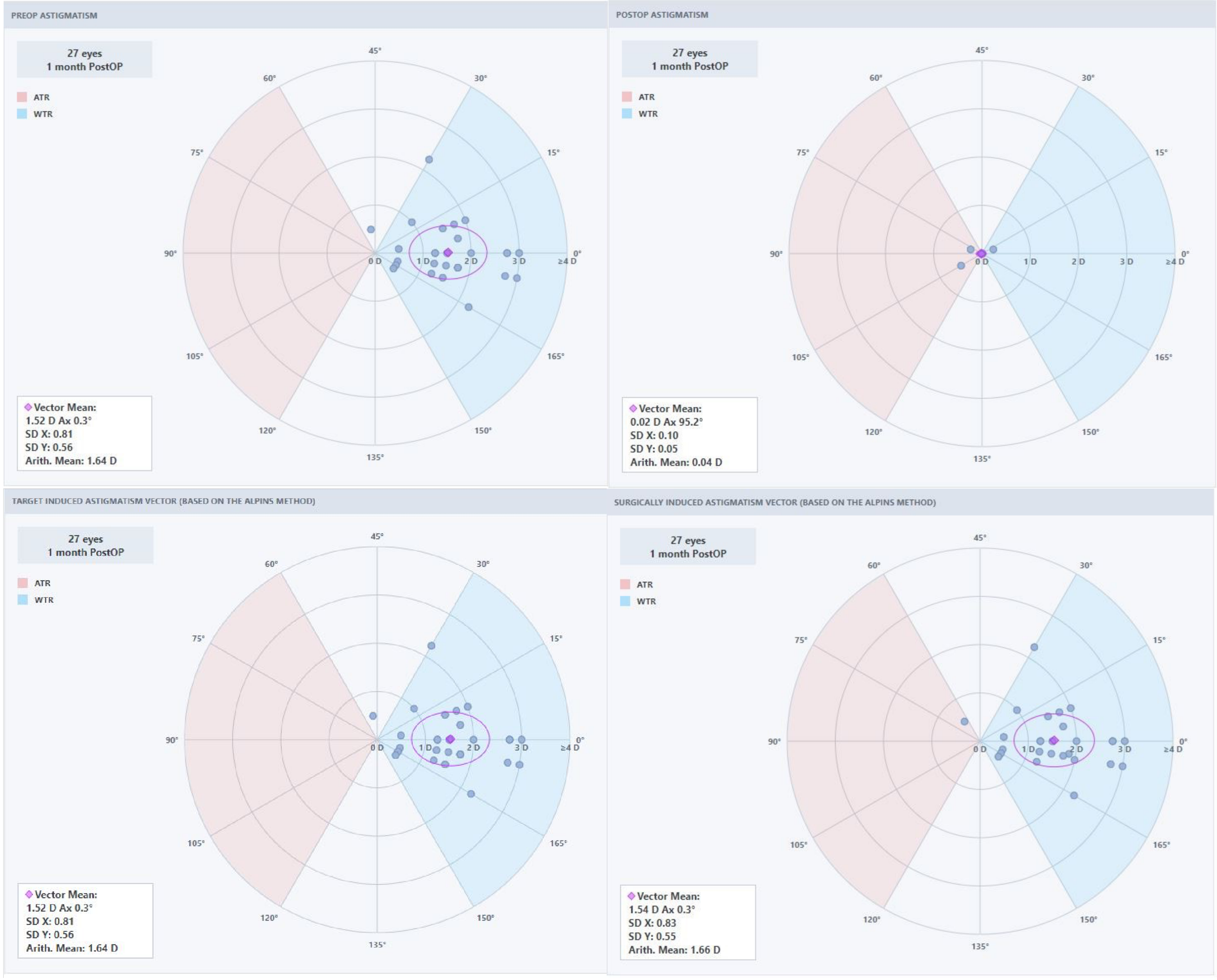
PREOP ASTIGMATISM
27 eyes
1 month PostOP
ATR
WTR
Vector Mean:
1.52 D Ax 0.3°
SD X: 0.81
SD Y: 0.56
Arith. Mean: 1.64 D
POSTOP ASTIGMATISM
27 eyes
1 month PostOP
ATR
WTR
Vector Mean:
0.02 D Ax 95.2°
SD X: 0.10
SD Y: 0.05
Arith. Mean: 0.04 D
TARGET INDUCED ASTIGMATISM VECTOR (BASED ON THE ALPINS METHOD)
27 eyes
1 month PostOP
ATR
WTR
Vector Mean:
1.52 D Ax 0.3°
SD X: 0.81
SD Y: 0.56
Arith. Mean: 1.64 D
SURGICALLY INDUCED ASTIGMATISM VECTOR (BASED ON THE ALPINS METHOD)
27 eyes
1 month PostOP
ATR
WTR
Vector Mean:
1.54 D Ax 0.3°
SD X: 0.83
SD Y: 0.55
Arith. Mean: 1.66 D

(C) OCULIGN
& NOMOGRAM

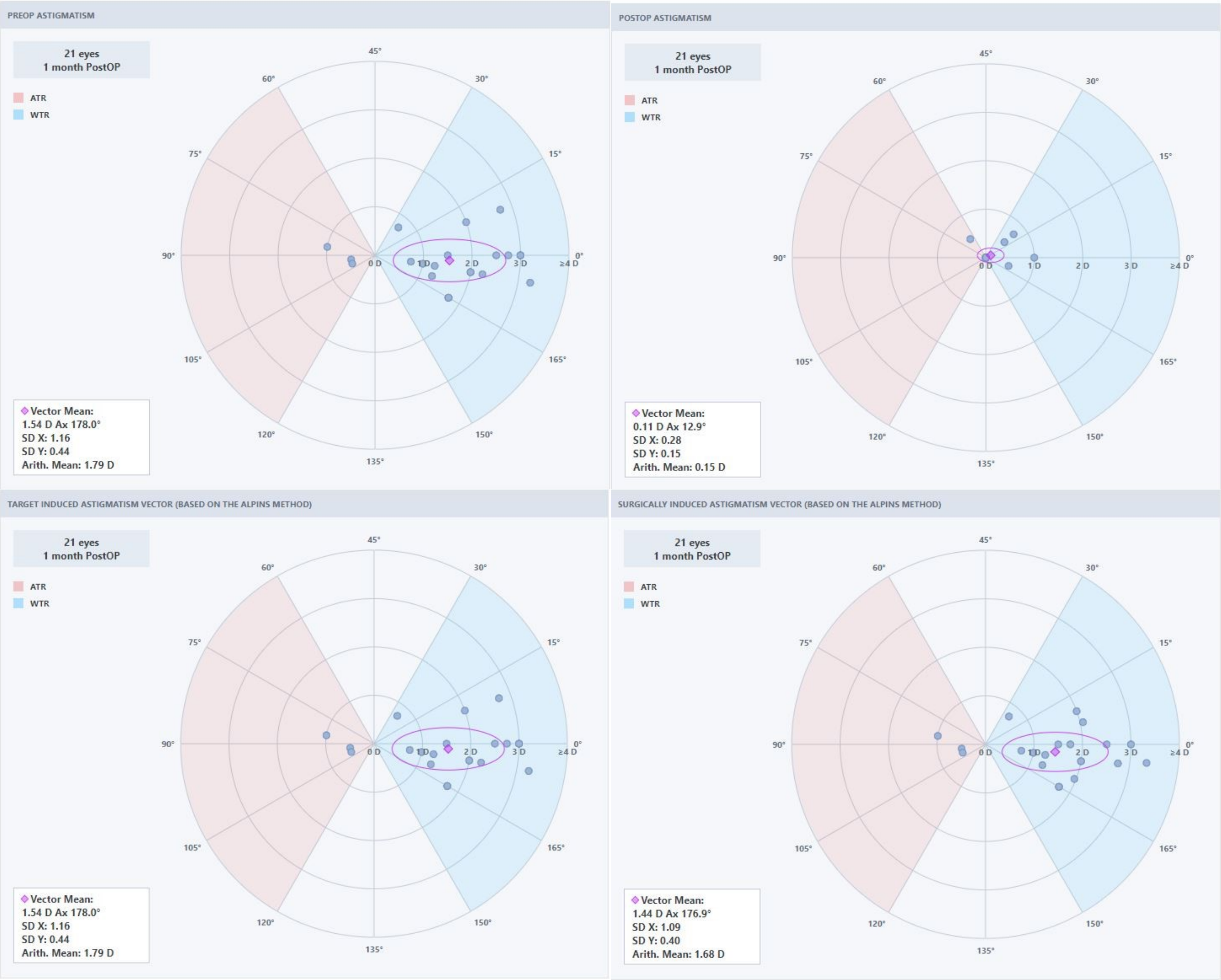

(D) OCULIGN

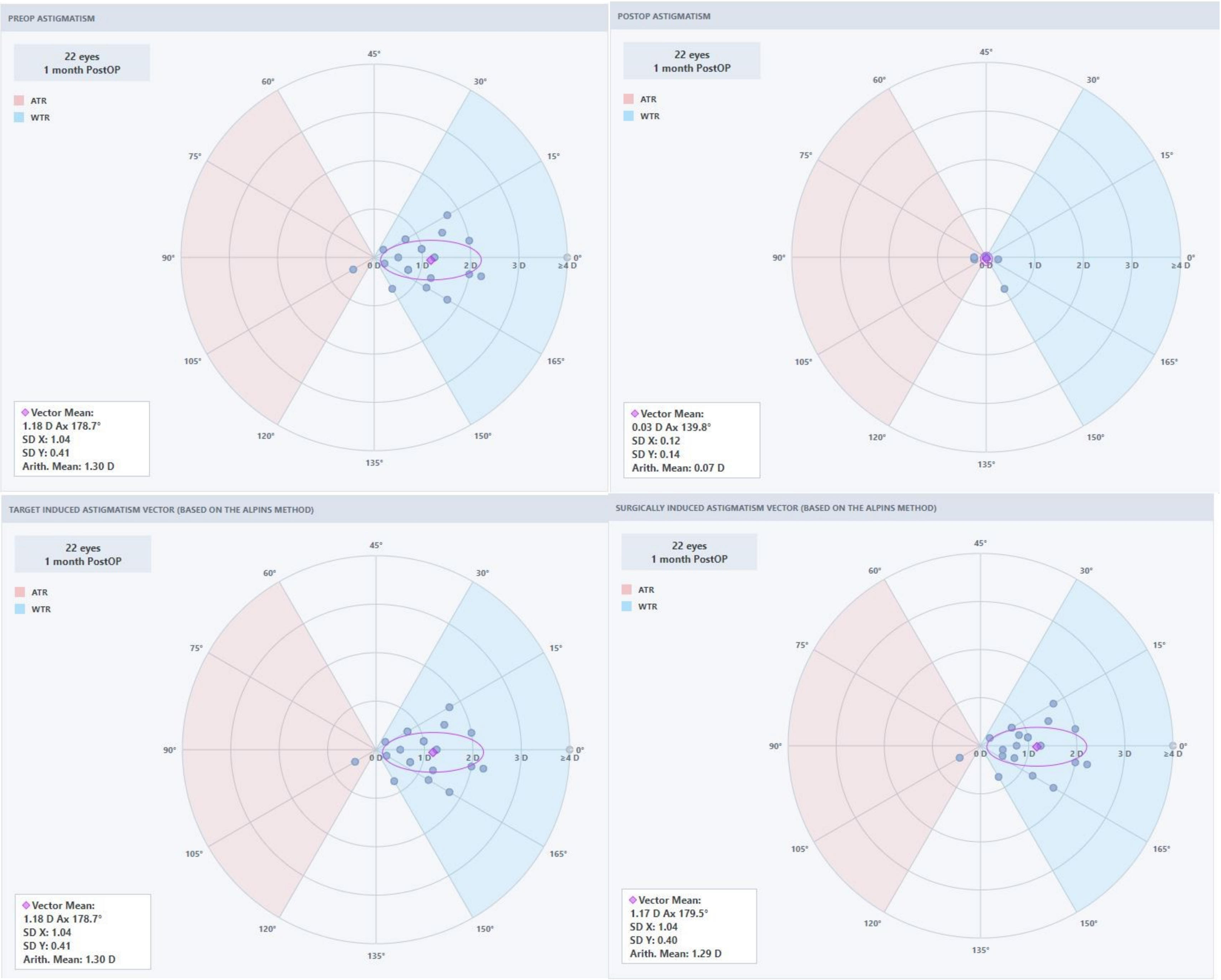

PREOP ASTIGMATISM
22 eyes
1 month PostOP
ATR
WTR
Vector Mean:
1.18 D Ax 178.7°
SD X: 1.04
SD Y: 0.41
Arith. Mean: 1.30 D
POSTOP ASTIGMATISM
22 eyes
1 month PostOP
ATR
WTR
Vector Mean:
0.03 D Ax 139.8°
SD X: 0.12
SD Y: 0.14
Arith. Mean: 0.07 D
TARGET INDUCED ASTIGMATISM VECTOR (BASED ON THE ALPINS METHOD)
22 eyes
1 month PostOP
ATR
WTR
Vector Mean:
1.18 D Ax 178.7°
SD X: 1.04
SD Y: 0.41
Arith. Mean: 1.30 D
SURGICALLY INDUCED ASTIGMATISM VECTOR (BASED ON THE ALPINS METHOD)
22 eyes
1 month PostOP
ATR
WTR
Vector Mean:
1.17 D Ax 179.5°
SD X: 1.04
SD Y: 0.40
Arith. Mean: 1.29 D
45°
60°
30°
75°
15°
90°
0°
0 D
1 D
2 D
3 D
≥4 D
105°
165°
120°
150°
135°
