# Supplementary material for: Utilising VISULYZE-Generated Nomograms and OcuLign Alignment Tools to Improve Keratorefractive Lenticule Extraction Outcomes
Source: J Clin Med. 2026 Apr 29;15(9):3389. doi: 10.3390/jcm15093389 (PMC13163256; doi:10.3390/jcm15093389)
Supplement: Supplementary file 1 [file jcm-15-03389-s001.zip › Revised Table S1.pdf]

# **Table S1.** Look-up table nomograms

| DEVICE TYPE | VISUMAX |
|---|---|
| TREATMENT TYPE | SMILE |
| KIND OF AMETROPIA | MYOPIC |

## ADJUSTED SPH/CYL CORRECTION (LASER)

| | SPH/CYL | SPH | | | | | | | | | | | | | | | |
|---|---|---|---|---|---|---|---|---|---|---|---|---|---|---|---|---|---|
| | | 0.00 | -0.25 | -0.50 | -0.75 | -1.00 | -1.25 | -1.50 | -1.75 | -2.00 | -2.25 | -2.50 | -2.75 | -3.00 | -3.25 | -3.50 | -3.75 |
| CYL | -0.00 | 0.00/0.00 | -0.26/0.00 | -0.52/0.00 | -0.78/0.00 | -1.04/0.00 | -1.30/0.00 | -1.57/0.00 | -1.83/0.00 | -2.09/0.00 | -2.35/0.00 | -2.61/0.00 | -2.87/0.00 | -3.13/0.00 | -3.39/0.00 | -3.65/0.00 | -3.91/0.00 |
| | -0.25 | -0.01/-0.25 | -0.27/-0.25 | -0.53/-0.25 | -0.79/-0.25 | -1.05/-0.25 | -1.31/-0.25 | -1.57/-0.25 | -1.83/-0.25 | -2.09/-0.25 | -2.35/-0.25 | -2.62/-0.25 | -2.88/-0.25 | -3.14/-0.25 | -3.40/-0.25 | -3.66/-0.25 | -3.92/-0.25 |
| | -0.50 | -0.01/-0.50 | -0.27/-0.50 | -0.54/-0.50 | -0.80/-0.50 | -1.06/-0.50 | -1.32/-0.50 | -1.58/-0.50 | -1.84/-0.50 | -2.10/-0.50 | -2.36/-0.50 | -2.62/-0.50 | -2.88/-0.50 | -3.14/-0.50 | -3.41/-0.50 | -3.67/-0.50 | -3.93/-0.50 |
| | -0.75 | -0.02/-0.74 | -0.28/-0.74 | -0.54/-0.74 | -0.80/-0.74 | -1.06/-0.74 | -1.32/-0.74 | -1.59/-0.74 | -1.85/-0.74 | -2.11/-0.74 | -2.37/-0.74 | -2.63/-0.74 | -2.89/-0.74 | -3.15/-0.74 | -3.41/-0.74 | -3.67/-0.74 | -3.93/-0.74 |
| | -1.00 | -0.03/-0.99 | -0.29/-0.99 | -0.55/-0.99 | -0.81/-0.99 | -1.07/-0.99 | -1.33/-0.99 | -1.59/-0.99 | -1.85/-0.99 | -2.11/-0.99 | -2.37/-0.99 | -2.64/-0.99 | -2.90/-0.99 | -3.16/-0.99 | -3.42/-0.99 | -3.68/-0.99 | -3.94/-0.99 |
| | -1.25 | -0.03/-1.24 | -0.29/-1.24 | -0.56/-1.24 | -0.82/-1.24 | -1.08/-1.24 | -1.34/-1.24 | -1.60/-1.24 | -1.86/-1.24 | -2.12/-1.24 | -2.38/-1.24 | -2.64/-1.24 | -2.90/-1.24 | -3.16/-1.24 | -3.43/-1.24 | -3.69/-1.24 | -3.95/-1.24 |
| | -1.50 | -0.04/-1.49 | -0.30/-1.49 | -0.56/-1.49 | -0.82/-1.49 | -1.08/-1.49 | -1.34/-1.49 | -1.61/-1.49 | -1.87/-1.49 | -2.13/-1.49 | -2.39/-1.49 | -2.65/-1.49 | -2.91/-1.49 | -3.17/-1.49 | -3.43/-1.49 | -3.69/-1.49 | -3.95/-1.49 |
| | -1.75 | -0.05/-1.73 | -0.31/-1.73 | -0.57/-1.73 | -0.83/-1.73 | -1.09/-1.73 | -1.35/-1.73 | -1.61/-1.73 | -1.87/-1.73 | -2.13/-1.73 | -2.39/-1.73 | -2.66/-1.73 | -2.92/-1.73 | -3.18/-1.73 | -3.44/-1.73 | -3.70/-1.73 | -3.96/-1.73 |
| | -2.00 | -0.05/-1.98 | -0.31/-1.98 | -0.57/-1.98 | -0.84/-1.98 | -1.10/-1.98 | -1.36/-1.98 | -1.62/-1.98 | -1.88/-1.98 | -2.14/-1.98 | -2.40/-1.98 | -2.66/-1.98 | -2.92/-1.98 | -3.18/-1.98 | -3.44/-1.98 | -3.71/-1.98 | -3.97/-1.98 |
| | -2.25 | -0.06/-2.23 | -0.32/-2.23 | -0.58/-2.23 | -0.84/-2.23 | -1.10/-2.23 | -1.36/-2.23 | -1.63/-2.23 | -1.89/-2.23 | -2.15/-2.23 | -2.41/-2.23 | -2.67/-2.23 | -2.93/-2.23 | -3.19/-2.23 | -3.45/-2.23 | -3.71/-2.23 | -3.97/-2.23 |
| | -2.50 | -0.07/-2.48 | -0.33/-2.48 | -0.59/-2.48 | -0.85/-2.48 | -1.11/-2.48 | -1.37/-2.48 | -1.63/-2.48 | -1.89/-2.48 | -2.15/-2.48 | -2.41/-2.48 | -2.68/-2.48 | -2.94/-2.48 | -3.20/-2.48 | -3.46/-2.48 | -3.72/-2.48 | -3.98/-2.48 |
| | -2.75 | -0.07/-2.72 | -0.33/-2.72 | -0.59/-2.72 | -0.86/-2.72 | -1.12/-2.72 | -1.38/-2.72 | -1.64/-2.72 | -1.90/-2.72 | -2.16/-2.72 | -2.42/-2.72 | -2.68/-2.72 | -2.94/-2.72 | -3.20/-2.72 | -3.46/-2.72 | -3.73/-2.72 | -3.99/-2.72 |
| | -3.00 | -0.08/-2.97 | -0.34/-2.97 | -0.60/-2.97 | -0.86/-2.97 | -1.12/-2.97 | -1.38/-2.97 | -1.65/-2.97 | -1.91/-2.97 | -2.17/-2.97 | -2.43/-2.97 | -2.69/-2.97 | -2.95/-2.97 | -3.21/-2.97 | -3.47/-2.97 | -3.73/-2.97 | -3.99/-2.97 |
| | -3.25 | -0.09/-3.22 | -0.35/-3.22 | -0.61/-3.22 | -0.87/-3.22 | -1.13/-3.22 | -1.39/-3.22 | -1.65/-3.22 | -1.91/-3.22 | -2.17/-3.22 | -2.43/-3.22 | -2.70/-3.22 | -2.96/-3.22 | -3.22/-3.22 | -3.48/-3.22 | -3.74/-3.22 | -4.00/-3.22 |
| | -3.50 | - | - | - | - | - | - | - | - | - | - | - | - | - | - | - | - |
| | -3.75 | - | - | - | - | - | - | - | - | - | - | - | - | - | - | - | - |
| | -4.00 | - | - | - | - | - | - | - | - | - | - | - | - | - | - | - | - |
| | -4.25 | - | - | - | - | - | - | - | - | - | - | - | - | - | - | - | - |
| | -4.50 | - | - | - | - | - | - | - | - | - | - | - | - | - | - | - | - |
| | -4.75 | - | - | - | - | - | - | - | - | - | - | - | - | - | - | - | - |
| | -5.00 | - | - | - | - | - | - | - | - | - | - | - | - | - | - | - | - |
| | -5.25 | - | - | - | - | - | - | - | - | - | - | - | - | - | - | - | - |
| | -5.50 | - | - | - | - | - | - | - | - | - | - | - | - | - | - | - | - |
| | -5.75 | - | - | - | - | - | - | - | - | - | - | - | - | - | - | - | - |
| | -6.00 | - | - | - | - | - | - | - | - | - | - | - | - | - | - | - | - |

| | |
|---|---|
| DEVICE TYPE | VISUMAX |
| TREATMENT TYPE | SMILE |
| KIND OF AMETROPIA | MYOPIC |

## ADJUSTED SPH/CYL CORRECTION (LASER)

| SPH/CYL | SPH | | | | | | | | | | | | | | | |
|---|---|---|---|---|---|---|---|---|---|---|---|---|---|---|---|---|
| CYL | -4.00 | -4.25 | -4.50 | -4.75 | -5.00 | -5.25 | -5.50 | -5.75 | -6.00 | -6.25 | -6.50 | -6.75 | -7.00 | -7.25 | -7.50 | -7.75 |
| -0.00 | -4.17/0.00 | -4.44/0.00 | -4.70/0.00 | -4.96/0.00 | -5.22/0.00 | -5.48/0.00 | -5.74/0.00 | -6.00/0.00 | -6.26/0.00 | -6.52/0.00 | -6.78/0.00 | -7.04/0.00 | -7.31/0.00 | -7.57/0.00 | -7.83/0.00 | -8.09/0.00 |
| -0.25 | -4.18/-0.25 | -4.44/-0.25 | -4.70/-0.25 | -4.96/-0.25 | -5.22/-0.25 | -5.49/-0.25 | -5.75/-0.25 | -6.01/-0.25 | -6.27/-0.25 | -6.53/-0.25 | -6.79/-0.25 | -7.05/-0.25 | -7.31/-0.25 | -7.57/-0.25 | -7.83/-0.25 | -8.10/-0.25 |
| -0.50 | -4.19/-0.50 | -4.45/-0.50 | -4.71/-0.50 | -4.97/-0.50 | -5.23/-0.50 | -5.49/-0.50 | -5.75/-0.50 | -6.01/-0.50 | -6.28/-0.50 | -6.54/-0.50 | -6.80/-0.50 | -7.06/-0.50 | -7.32/-0.50 | -7.58/-0.50 | -7.84/-0.50 | -8.10/-0.50 |
| -0.75 | -4.19/-0.74 | -4.46/-0.74 | -4.72/-0.74 | -4.98/-0.74 | -5.24/-0.74 | -5.50/-0.74 | -5.76/-0.74 | -6.02/-0.74 | -6.28/-0.74 | -6.54/-0.74 | -6.80/-0.74 | -7.06/-0.74 | -7.33/-0.74 | -7.59/-0.74 | -7.85/-0.74 | -8.11/-0.74 |
| -1.00 | -4.20/-0.99 | -4.46/-0.99 | -4.72/-0.99 | -4.98/-0.99 | -5.24/-0.99 | -5.51/-0.99 | -5.77/-0.99 | -6.03/-0.99 | -6.29/-0.99 | -6.55/-0.99 | -6.81/-0.99 | -7.07/-0.99 | -7.33/-0.99 | -7.59/-0.99 | -7.85/-0.99 | -8.11/-0.99 |
| -1.25 | -4.21/-1.24 | -4.47/-1.24 | -4.73/-1.24 | -4.99/-1.24 | -5.25/-1.24 | -5.51/-1.24 | -5.77/-1.24 | -6.03/-1.24 | -6.30/-1.24 | -6.56/-1.24 | -6.82/-1.24 | -7.08/-1.24 | -7.34/-1.24 | -7.60/-1.24 | -7.86/-1.24 | -8.12/-1.24 |
| -1.50 | -4.21/-1.49 | -4.48/-1.49 | -4.74/-1.49 | -5.00/-1.49 | -5.26/-1.49 | -5.52/-1.49 | -5.78/-1.49 | -6.04/-1.49 | -6.30/-1.49 | -6.56/-1.49 | -6.82/-1.49 | -7.08/-1.49 | -7.35/-1.49 | -7.61/-1.49 | -7.87/-1.49 | -8.13/-1.49 |
| -1.75 | -4.22/-1.73 | -4.48/-1.73 | -4.74/-1.73 | -5.00/-1.73 | -5.26/-1.73 | -5.53/-1.73 | -5.79/-1.73 | -6.05/-1.73 | -6.31/-1.73 | -6.57/-1.73 | -6.83/-1.73 | -7.09/-1.73 | -7.35/-1.73 | -7.61/-1.73 | -7.87/-1.73 | -8.13/-1.73 |
| -2.00 | -4.23/-1.98 | -4.49/-1.98 | -4.75/-1.98 | -5.01/-1.98 | -5.27/-1.98 | -5.53/-1.98 | -5.79/-1.98 | -6.05/-1.98 | -6.32/-1.98 | -6.58/-1.98 | -6.84/-1.98 | -7.10/-1.98 | -7.36/-1.98 | -7.62/-1.98 | -7.88/-1.98 | -8.14/-1.98 |
| -2.25 | -4.23/-2.23 | -4.50/-2.23 | -4.76/-2.23 | -5.02/-2.23 | -5.28/-2.23 | -5.54/-2.23 | -5.80/-2.23 | -6.06/-2.23 | -6.32/-2.23 | -6.58/-2.23 | -6.84/-2.23 | -7.10/-2.23 | -7.37/-2.23 | -7.63/-2.23 | -7.89/-2.23 | -8.15/-2.23 |
| -2.50 | -4.24/-2.48 | -4.50/-2.48 | -4.76/-2.48 | -5.02/-2.48 | -5.28/-2.48 | -5.55/-2.48 | -5.81/-2.48 | -6.07/-2.48 | -6.33/-2.48 | -6.59/-2.48 | -6.85/-2.48 | -7.11/-2.48 | -7.37/-2.48 | -7.63/-2.48 | -7.89/-2.48 | -8.15/-2.48 |
| -2.75 | -4.25/-2.72 | -4.51/-2.72 | -4.77/-2.72 | -5.03/-2.72 | -5.29/-2.72 | -5.55/-2.72 | -5.81/-2.72 | -6.07/-2.72 | -6.33/-2.72 | -6.60/-2.72 | -6.86/-2.72 | -7.12/-2.72 | -7.38/-2.72 | -7.64/-2.72 | -7.90/-2.72 | -8.16/-2.72 |
| -3.00 | -4.25/-2.97 | -4.52/-2.97 | -4.78/-2.97 | -5.04/-2.97 | -5.30/-2.97 | -5.56/-2.97 | -5.82/-2.97 | -6.08/-2.97 | -6.34/-2.97 | -6.60/-2.97 | -6.86/-2.97 | -7.12/-2.97 | -7.39/-2.97 | -7.65/-2.97 | -7.91/-2.97 | -8.17/-2.97 |
| -3.25 | -4.26/-3.22 | -4.52/-3.22 | -4.78/-3.22 | -5.04/-3.22 | -5.30/-3.22 | -5.57/-3.22 | -5.83/-3.22 | -6.09/-3.22 | -6.35/-3.22 | -6.61/-3.22 | -6.87/-3.22 | -7.13/-3.22 | -7.39/-3.22 | -7.65/-3.22 | -7.91/-3.22 | -8.17/-3.22 |
| -3.50 | - | - | - | - | - | - | - | - | - | - | - | - | - | - | - | - |
| -3.75 | - | - | - | - | - | - | - | - | - | - | - | - | - | - | - | - |
| -4.00 | - | - | - | - | - | - | - | - | - | - | - | - | - | - | - | - |
| -4.25 | - | - | - | - | - | - | - | - | - | - | - | - | - | - | - | - |
| -4.50 | - | - | - | - | - | - | - | - | - | - | - | - | - | - | - | - |
| -4.75 | - | - | - | - | - | - | - | - | - | - | - | - | - | - | - | - |
| -5.00 | - | - | - | - | - | - | - | - | - | - | - | - | - | - | - | - |
| -5.25 | - | - | - | - | - | - | - | - | - | - | - | - | - | - | - | - |
| -5.50 | - | - | - | - | - | - | - | - | - | - | - | - | - | - | - | - |
| -5.75 | - | - | - | - | - | - | - | - | - | - | - | - | - | - | - | - |
| -6.00 | - | - | - | - | - | - | - | - | - | - | - | - | - | - | - | - |

| DEVICE TYPE | VISUMAX |
|---|---|
| TREATMENT TYPE | SMILE |
| KIND OF AMETROPIA | MYOPIC |

## ADJUSTED SPH/CYL CORRECTION (LASER)

| SPH/CYL | SPH | | | | | | | | | | | | | | | |
|---|---|---|---|---|---|---|---|---|---|---|---|---|---|---|---|---|
| | -4.00 | -4.25 | -4.50 | -4.75 | -5.00 | -5.25 | -5.50 | -5.75 | -6.00 | -6.25 | -6.50 | -6.75 | -7.00 | -7.25 | -7.50 | -7.75 |
| -0.00 | -4.17/0.00 | -4.44/0.00 | -4.70/0.00 | -4.96/0.00 | -5.22/0.00 | -5.48/0.00 | -5.74/0.00 | -6.00/0.00 | -6.26/0.00 | -6.52/0.00 | -6.78/0.00 | -7.04/0.00 | -7.31/0.00 | -7.57/0.00 | -7.83/0.00 | -8.09/0.00 |
| -0.25 | -4.18/-0.25 | -4.44/-0.25 | -4.70/-0.25 | -4.96/-0.25 | -5.22/-0.25 | -5.49/-0.25 | -5.75/-0.25 | -6.01/-0.25 | -6.27/-0.25 | -6.53/-0.25 | -6.79/-0.25 | -7.05/-0.25 | -7.31/-0.25 | -7.57/-0.25 | -7.83/-0.25 | -8.10/-0.25 |
| -0.50 | -4.19/-0.50 | -4.45/-0.50 | -4.71/-0.50 | -4.97/-0.50 | -5.23/-0.50 | -5.49/-0.50 | -5.75/-0.50 | -6.01/-0.50 | -6.28/-0.50 | -6.54/-0.50 | -6.80/-0.50 | -7.06/-0.50 | -7.32/-0.50 | -7.58/-0.50 | -7.84/-0.50 | -8.10/-0.50 |
| -0.75 | -4.19/-0.74 | -4.46/-0.74 | -4.72/-0.74 | -4.98/-0.74 | -5.24/-0.74 | -5.50/-0.74 | -5.76/-0.74 | -6.02/-0.74 | -6.28/-0.74 | -6.54/-0.74 | -6.80/-0.74 | -7.06/-0.74 | -7.33/-0.74 | -7.59/-0.74 | -7.85/-0.74 | -8.11/-0.74 |
| -1.00 | -4.20/-0.99 | -4.46/-0.99 | -4.72/-0.99 | -4.98/-0.99 | -5.24/-0.99 | -5.51/-0.99 | -5.77/-0.99 | -6.03/-0.99 | -6.29/-0.99 | -6.55/-0.99 | -6.81/-0.99 | -7.07/-0.99 | -7.33/-0.99 | -7.59/-0.99 | -7.85/-0.99 | -8.11/-0.99 |
| -1.25 | -4.21/-1.24 | -4.47/-1.24 | -4.73/-1.24 | -4.99/-1.24 | -5.25/-1.24 | -5.51/-1.24 | -5.77/-1.24 | -6.03/-1.24 | -6.30/-1.24 | -6.56/-1.24 | -6.82/-1.24 | -7.08/-1.24 | -7.34/-1.24 | -7.60/-1.24 | -7.86/-1.24 | -8.12/-1.24 |
| -1.50 | -4.21/-1.49 | -4.48/-1.49 | -4.74/-1.49 | -5.00/-1.49 | -5.26/-1.49 | -5.52/-1.49 | -5.78/-1.49 | -6.04/-1.49 | -6.30/-1.49 | -6.56/-1.49 | -6.82/-1.49 | -7.08/-1.49 | -7.35/-1.49 | -7.61/-1.49 | -7.87/-1.49 | -8.13/-1.49 |
| -1.75 | -4.22/-1.73 | -4.48/-1.73 | -4.74/-1.73 | -5.00/-1.73 | -5.26/-1.73 | -5.53/-1.73 | -5.79/-1.73 | -6.05/-1.73 | -6.31/-1.73 | -6.57/-1.73 | -6.83/-1.73 | -7.09/-1.73 | -7.35/-1.73 | -7.61/-1.73 | -7.87/-1.73 | -8.13/-1.73 |
| -2.00 | -4.23/-1.98 | -4.49/-1.98 | -4.75/-1.98 | -5.01/-1.98 | -5.27/-1.98 | -5.53/-1.98 | -5.79/-1.98 | -6.05/-1.98 | -6.32/-1.98 | -6.58/-1.98 | -6.84/-1.98 | -7.10/-1.98 | -7.36/-1.98 | -7.62/-1.98 | -7.88/-1.98 | -8.14/-1.98 |
| -2.25 | -4.23/-2.23 | -4.50/-2.23 | -4.76/-2.23 | -5.02/-2.23 | -5.28/-2.23 | -5.54/-2.23 | -5.80/-2.23 | -6.06/-2.23 | -6.32/-2.23 | -6.58/-2.23 | -6.84/-2.23 | -7.10/-2.23 | -7.37/-2.23 | -7.63/-2.23 | -7.89/-2.23 | -8.15/-2.23 |
| -2.50 | -4.24/-2.48 | -4.50/-2.48 | -4.76/-2.48 | -5.02/-2.48 | -5.28/-2.48 | -5.55/-2.48 | -5.81/-2.48 | -6.07/-2.48 | -6.33/-2.48 | -6.59/-2.48 | -6.85/-2.48 | -7.11/-2.48 | -7.37/-2.48 | -7.63/-2.48 | -7.89/-2.48 | -8.15/-2.48 |
| -2.75 | -4.25/-2.72 | -4.51/-2.72 | -4.77/-2.72 | -5.03/-2.72 | -5.29/-2.72 | -5.55/-2.72 | -5.81/-2.72 | -6.07/-2.72 | -6.33/-2.72 | -6.60/-2.72 | -6.86/-2.72 | -7.12/-2.72 | -7.38/-2.72 | -7.64/-2.72 | -7.90/-2.72 | -8.16/-2.72 |
| CYL -3.00 | -4.25/-2.97 | -4.52/-2.97 | -4.78/-2.97 | -5.04/-2.97 | -5.30/-2.97 | -5.56/-2.97 | -5.82/-2.97 | -6.08/-2.97 | -6.34/-2.97 | -6.60/-2.97 | -6.86/-2.97 | -7.12/-2.97 | -7.39/-2.97 | -7.65/-2.97 | -7.91/-2.97 | -8.17/-2.97 |
| -3.25 | -4.26/-3.22 | -4.52/-3.22 | -4.78/-3.22 | -5.04/-3.22 | -5.30/-3.22 | -5.57/-3.22 | -5.83/-3.22 | -6.09/-3.22 | -6.35/-3.22 | -6.61/-3.22 | -6.87/-3.22 | -7.13/-3.22 | -7.39/-3.22 | -7.65/-3.22 | -7.91/-3.22 | -8.17/-3.22 |
| -3.50 | - | - | - | - | - | - | - | - | - | - | - | - | - | - | - | - |
| -3.75 | - | - | - | - | - | - | - | - | - | - | - | - | - | - | - | - |
| -4.00 | - | - | - | - | - | - | - | - | - | - | - | - | - | - | - | - |
| -4.25 | - | - | - | - | - | - | - | - | - | - | - | - | - | - | - | - |
| -4.50 | - | - | - | - | - | - | - | - | - | - | - | - | - | - | - | - |
| -4.75 | - | - | - | - | - | - | - | - | - | - | - | - | - | - | - | - |
| -5.00 | - | - | - | - | - | - | - | - | - | - | - | - | - | - | - | - |
| -5.25 | - | - | - | - | - | - | - | - | - | - | - | - | - | - | - | - |
| -5.50 | - | - | - | - | - | - | - | - | - | - | - | - | - | - | - | - |
| -5.75 | - | - | - | - | - | - | - | - | - | - | - | - | - | - | - | - |
| -6.00 | - | - | - | - | - | - | - | - | - | - | - | - | - | - | - | - |

| | |
|---|---|
| DEVICE TYPE | VISUMAX |
| TREATMENT TYPE | SMILE |
| KIND OF AMETROPIA | MYOPIC |

## ADJUSTED SPH/CYL CORRECTION (LASER)

| SPH/CYL | SPH | | | | | | | | | | | | | | | |
|---|---|---|---|---|---|---|---|---|---|---|---|---|---|---|---|---|
| CYL | -12.00 | -12.25 | -12.50 | -12.75 | -13.00 | -13.25 | -13.50 | -13.75 | -14.00 | -14.25 | -14.50 | -14.75 | -15.00 | -15.25 | -15.50 | -15.75 |
| -0.00 | - | - | - | - | - | - | - | - | - | - | - | - | - | - | - | - |
| -0.25 | - | - | - | - | - | - | - | - | - | - | - | - | - | - | - | - |
| -0.50 | - | - | - | - | - | - | - | - | - | - | - | - | - | - | - | - |
| -0.75 | - | - | - | - | - | - | - | - | - | - | - | - | - | - | - | - |
| -1.00 | - | - | - | - | - | - | - | - | - | - | - | - | - | - | - | - |
| -1.25 | - | - | - | - | - | - | - | - | - | - | - | - | - | - | - | - |
| -1.50 | - | - | - | - | - | - | - | - | - | - | - | - | - | - | - | - |
| -1.75 | - | - | - | - | - | - | - | - | - | - | - | - | - | - | - | - |
| -2.00 | - | - | - | - | - | - | - | - | - | - | - | - | - | - | - | - |
| -2.25 | - | - | - | - | - | - | - | - | - | - | - | - | - | - | - | - |
| -2.50 | - | - | - | - | - | - | - | - | - | - | - | - | - | - | - | - |
| -2.75 | - | - | - | - | - | - | - | - | - | - | - | - | - | - | - | - |
| -3.00 | - | - | - | - | - | - | - | - | - | - | - | - | - | - | - | - |
| -3.25 | - | - | - | - | - | - | - | - | - | - | - | - | - | - | - | - |
| -3.50 | - | - | - | - | - | - | - | - | - | - | - | - | - | - | - | - |
| -3.75 | - | - | - | - | - | - | - | - | - | - | - | - | - | - | - | - |
| -4.00 | - | - | - | - | - | - | - | - | - | - | - | - | - | - | - | - |
| -4.25 | - | - | - | - | - | - | - | - | - | - | - | - | - | - | - | - |
| -4.50 | - | - | - | - | - | - | - | - | - | - | - | - | - | - | - | - |
| -4.75 | - | - | - | - | - | - | - | - | - | - | - | - | - | - | - | - |
| -5.00 | - | - | - | - | - | - | - | - | - | - | - | - | - | - | - | - |
| -5.25 | - | - | - | - | - | - | - | - | - | - | - | - | - | - | - | - |
| -5.50 | - | - | - | - | - | - | - | - | - | - | - | - | - | - | - | - |
| -5.75 | - | - | - | - | - | - | - | - | - | - | - | - | - | - | - | - |
| -6.00 | - | - | - | - | - | - | - | - | - | - | - | - | - | - | - | - |
