# Supplementary material for: Utilising VISULYZE-Generated Nomograms and OcuLign Alignment Tools to Improve Keratorefractive Lenticule Extraction Outcomes
Source: J Clin Med. 2026 Apr 29;15(9):3389. doi: 10.3390/jcm15093389 (PMC13163256; doi:10.3390/jcm15093389)
Supplement: Supplementary file 1 [file jcm-15-03389-s001.zip › Revised Table S2.pdf]

Table S2. Intra-operative parameters

Intra-operative Parameters	PRE-NOMOGRAM (N = 378 Eyes, 100%)	POST-NOMOGRAM (N = 425 Eyes, 100%)	OCULIGN & NOMOGRAM (N = 212 Eyes, 100%)	OCULIGN (N = 249 Eyes, 100%)	*p*-Value
Cap thickness	107.65 ± 4.432 (100 to 120)	108.14 ± 4.406 (100 to 120)	107.78 ± 4.699 (100 to 120)	107.31 ± 4.958 (100 to 120)	0.122 [b]
Optical zone	6.253 ± 0.1985 (5.8 to 7.0)	6.273 ± 0.1781 (5.6 to 7.0)	6.311 ± 0.2271 (5.8 to 7.0)	6.323 ± 0.2814 (5.8 to 7.0)	<0.001 [b*]
PX	0.011 ± 0.1877 (−0.5 to 0.6)	0.003 ± 0.1687 (−0.6 to 0.5)	0.017 ± 0.2722 (−0.5 to 3.0)	0.007 ± 0.2890 (−2.0 to 3.0)	0.964 [b]
PY	−0.001 ± 0.1497 (−0.5 to 0.4)	−0.006 ± 0.1390 (−0.4 to 0.5)	0.002 ± 0.1497 (−0.5 to 1.0)	−0.002 ± 0.1320 (−0.4 to 0.4)	0.921 [b]
AX	−0.007 ± 0.1699 (-0.6 to 0.4)	−0.007 ± 0.1689 (−0.4 to 0.5)	0.017 ± 0.3486 (−0.5 to 4.0)	−0.030 ± 0.2497 (−2.0 to 0.5)	0.783 [b]
AY	−0.035 ± 0.1314 (−0.4 to 0.3)	−0.026 ± 0.1306 (−0.4 to 0.4)	−0.045 ± 0.1571 (−0.5 to 1.0)	−0.020 ± 0.2358 (−3.0 to 1.0)	0.029 [b*]
Decentring	0.151 ± 0.0959 (0 to 0.7)	0.153 ± 0.0924 (0 to 0.5)	0.129 ± 0.0953 (0 to 1.0)	0.167 ± 0.1015 (0 to 1.0)	<0.001 [b*]
Energy index	27 ± 0 (27)	26.84 ± 0.0924 (26 to 27)	26.99 ± 0.118 (26 to 27)	25.96 ± 0.563 (24 to 27)	<0.001 [b*]
Spot distance	4.199 ± 0.0073 (4.1 to 4.2)	4.002 ± 0.4456 (3.0 to 4.2)	4.183 ± 0.1421 (3.0 to 4.2)	3.154 ± 0.4024 (3.0 to 4.2)	<0.001 [b*]
Track distance	4.0 ± 0 (4.0)	3.835 ± 0.3714 (3.0 to 4.0)	3.986 ± 0.1184 (3.0 to 4.0)	3.138 ± 0.3622 (3.0 to 4.2)	<0.001 [b*]
Cyclotorsion	0	0	−0.45 ± 3.761 (−11 to 10)	0.02 ± 3.990 (−10 to 10)	0.040 [b*]
SPH adjustment	N/A	5.20 ± 0.916 (0 to 11)	5.26 ± 0.961 (1 to 10)	0	<0.001 [b*]
CYL adjustment	N/A	−0.97 ± 0.238 (−1 to 1)	−0.99 ± 0.137 (−1 to 1)	0	<0.001 [b*]
SPH laser setting	N/A	-5.349 ± 2.083 (−9.965 to 0.549)	−4.917 ± 2.399 (−10.70 to −0.542)	−4.88 ± 2.435 (−10.00 to −0.50)	0.091 [b]
CYL laser setting	N/A	−1.25 ± 0.735 (−4.031 to 0)	−1.17 ± 0.835 (−3.217 to 2.722)	−1.55 ± 1.139 (−5.00 to 2.50)	<0.001 [b*]
Oculign cyclotorsion	N/A	N/A	−0.50 ± 3.471 (−11 to 10)	0 ± 3.987 (−10 to 10)	0.193 [b]
Oculign confidence interval	N/A	N/A	0.89 ± 0.13 (0.33 to 0.98)	0.85 ± 0.16 (0.33 to 0.98)	0.005 [b*]

*Statistically significant, *p*-value<0.05; [b] Using Kruskal-Wallis test; AX, achieved centration x; AY, achieved centration y; LE, left eye; N/A, not available; PX, centration target position x; PY, centration target position y; RE, right eye; RST, residual stromal thickness.
